# Two new *Lactarius* species from Laos (*Basidiomycota*, *Russulales*)

**DOI:** 10.3897/mycokeys.132.186093

**Published:** 2026-05-08

**Authors:** Annemieke Verbeken, Ole S. Pedersen, Andy F. S. Taylor, Jorinde Nuytinck

**Affiliations:** 1 Dpt. Biology, Ghent University, K.L. Ledeganckstraat 35, B-9000 Ghent, Belgium Dpt. Biology, Ghent University Ghent Belgium; 2 120/1-2 Sukhumvit 49, 10110 Wattana, Bangkok, Thailand School of Biological Sciences, University of Aberdeen Aberdeen United Kingdom; 3 The James Hutton Institute, Craigiebuckler, Aberdeen, AB15 8QH, UK Naturalis Biodiversity Center Leiden Netherlands; 4 School of Biological Sciences, University of Aberdeen, Aberdeen, AB24 3UUuel, UK Unaffiliated Bangkok Thailand; 5 Naturalis Biodiversity Center, PO Box 9517, 2300 RA Leiden, Netherlands The James Hutton Institute Aberdeen United Kingdom

**Keywords:** *
Fagaceae
*, ITS phylogeny, *
Lactarius
*, microscopy, *Pinus kesiya*, Southeast Asia

## Abstract

Two new species of milkcaps, *Lactarius megaplinthogalus* and *L. rosascens*, are described from montane forests in Xieng Khouang Province, northern Laos, Southeast Asia. Both species were collected in mixed forests dominated by *Fagaceae* and *Pinaceae* and are documented using detailed macro- and micromorphological observations in combination with ITS rDNA sequence data. *Lactarius megaplinthogalus* is distinguished by its large, stout, dark brown to blackish basidiocarps, very distant lamellae, sticky white latex that stains the lamellae pinkish to blackish, and thick-walled basidia. *Lactarius rosascens* is recognized by its yellow to honey-colored, scrobiculate pileus, abundant white latex rapidly turning bright pink to wine-red and intensely staining the lamellae, and a well-developed trichodermal pileipellis. Phylogenetic analyses support their recognition as distinct species and place the first species in *L.* subg. *Plinthogalus* (section *Plinthogalus*) and the latter one in *L.* subg. *Lactarius* (unclassified at the sectional level). These findings highlight the exceptional and still underexplored diversity of milkcaps in Laos (and more generally in Southeast Asia) and underline the importance of continued mycological surveys and integrative taxonomic approaches in the region.

## Introduction

With an estimated 90% of fungal species still unknown to science, the challenges of uncovering hidden fungal diversity are immense, not only for microfungi with cryptic lifestyles but even for fungi that produce large and conspicuous fruitbodies (Hyde et al. 2024; Hongsanan et al. 2025; Nie et al. 2025). Tropical regions, including Southeast Asia, are recognized as biodiversity hotspots for macrofungi, yet they remain far less explored than temperate regions (Mueller et al. 2007; Stallman et al. 2024; Zhao et al. 2026). Laos, with its rich and diverse forest ecosystems, has a forest cover of approximately 58% (MAF 2021). Dipterocarp-rich lowland forests (400–800 m.a.s.l.) dominate the central and southern parts of the country, while montane forests (above 1000 m.a.s.l.) occur mainly in the north (MoNRE 2016). The northern mountainous Xieng Khouang Province, located between 500 and nearly 2,300 m.a.s.l., harbors a wide range of forest types, including pine plantations, mixed conifer-broadleaved forests, moist and dry evergreen forests, riverine and swamp forests, dry deciduous forests on limestone, and moist, dense, and open secondary forests (Lehmann et al. 2003).

Ectomycorrhizal host trees include species in *Betulaceae (Betula)*, *Fagaceae* (*Castanopsis*, *Lithocarpus*, and *Quercus*), *Leguminosae (Ormosia)*, and *Pinaceae* (*Keteleeria* and *Pinus*; PBSAP 2013). Consequently, Laos in general and Xieng Khouang Province in particular offer substantial untapped potential for mycological discovery.

The relatively low level of fungal exploration in the country is largely influenced by typical subtropical to tropical climatic conditions and complex topography, which make many areas difficult to access and work in. At the same time, the remoteness of these forests has helped safeguard their ecological value. A further limiting factor is the lack of trained mycologists.

In this context, a DEFRA-funded Darwin Initiative project (project number 21-002) that ran from 2014–2017 focused on enhancing mycological research and capacity building in Laos and aimed to improve knowledge of fungal biodiversity and support the sustainable management of fungal resources in the region. Fieldwork conducted within the framework of this training project, as well as its collaboration project, the UNDP-FAO Agrobiodiversity Project (2011–2017), has so far resulted in the description of 10 new species in the genera *Leucoagaricus*, *Hebeloma*, *Cystolepiota*, *Amanita*, and *Nigrocarnea* (Sysouphanthong et al. 2018, 2022; Eberhardt et al. 2020; van de Peppel et al. 2022; Sysouphanthong and Thongklang 2022; Manawasinghe et al. 2022; Pedersen and Læssøe 2024). Several additional taxa are currently awaiting taxonomic treatment in the fungarium.

Especially in tropical regions, numerous milkcap species formerly assigned to *Lactarius* have been transferred to *Lactifluus*, following molecular phylogenetic evidence showing that the traditional concept of *Lactarius* does not represent a monophyletic genus (Buyck et al. 2008, 2010). Some milkcap records from Laos correspond to *Lactifluus* species now, even though older literature might list them as *Lactarius* (Van de Putte et al. 2010). For differences between the milkcap genera *Lactarius* and *Lactifluus*, we refer to Verbeken and Nuytinck (2013).

No former study in Laos has focused on *Lactariussensu stricto*. Current knowledge of *Lactarius* diversity in tropical to subtropical Asia is largely based on regional and taxon-focused surveys conducted in East and Southeast Asia (e.g., China, Thailand, Vietnam), as well as in the Himalayan–Hengduan montane regions of temperate to subtropical Asia (Le et al. 2007a, 2007b, 2007c; Wang et al. 2008, 2012, 2025; Stubbe et al. 2012; Verbeken et al. 2014a, 2014b; Wisitrassameewong et al. 2014a, 2014b, 2015; De Crop et al. 2018a, 2018b; Tang et al. 2022; Huang et al. 2022). These studies have highlighted the exceptional diversity of Asian milkcaps and their distinctive morphological and ecological adaptations in subtropical to tropical forest ecosystems.

Here, we introduce two new and striking species of milkcaps *sensu stricto*, both belonging to the genus *Lactarius*, using morphological and phylogenetic features.

## Material and methods

### Morphological study

Macroscopic characters are all based on fresh material. Colors are described according to Kornerup and Wanscher (1978). Microscopic features were studied from dried material mainly in Congo red in L4. Spore ornamentation is described and illustrated as observed in Melzer’s reagent. For details on terminology, we refer to Verbeken (1998) and Verbeken and Walleyn (2010). Line drawings were made by A. Verbeken with the aid of a drawing tube at original magnifications: 6000 × for spores, 1000 × for individual elements and sections. Basidia length excludes sterigmata length. Spores were measured in side view in Melzer’s reagent, excluding the ornamentation, and measurements are given as {(MIN) [AV-2*SD] – AV– [AV+2*SD] (MAX)}_length_ x {(MIN) [AV-2*SD] – AV– [AV+2*SD] (MAX)}_width,_ in which AV = mean value for the measured collection and SD = standard deviation. *Q* stands for “quotient length/width” and is given as MINQ – AvQ – MAXQ, in which AvQ stands for the mean quotient for the measured spores.

Dried specimens are conserved in the following fungaria: National Herbarium of Laos (HNL) and Herbarium Universitatis Gandavensis partim Mycology (GENT).

### DNA extraction, PCR amplification, and sequencing

DNA was extracted from fresh material stored in 2×CTAB buffer during the fieldwork. DNA was extracted manually with the CTAB method (Nuytinck and Verbeken 2003, modified by Van de Putte et al. 2010) or in a high-throughput fashion. The high-throughput method involved material lysis with a TissueLyser (Qiagen, Hilden, Germany) and DNA purification using a KingFisher™ extraction robot (Thermo Scientific, Waltham, MA, USA) with magnetic particle separation and the NucleoMag Plant kit (Machery-Nagel, Düren, Germany). The internal transcribed spacer region of the nuclear ribosomal DNA (ITS) was amplified and sequenced using primers ITS1-F and ITS4 (White et al. 1990; Gardes and Bruns 1993). In a final volume of 25 μL, 2.5 μL of 10× CoralLoad buffer (Qiagen, Hilden, Germany), 1 μL of each primer (10 μM), 1 μL dNTPs (2.5 mM), 1.5 μL MgCl_2_ (2.5 mM), 0.25 μL Taq polymerase (5 U/μL, Qiagen), and 1 μL of DNA template were mixed. Reaction mixtures were preheated at 96 °C for 5 min, followed by 40 cycles of denaturation at 96 °C for 45 sec, primer annealing at 45 °C for 45 sec, and elongation at 72 °C for 60 sec, with final extension at 72 °C for 7 min. PCR amplification success was checked on an E-Gel™ with SYBR™ Safe DNA Gel Stain, 2% (Invitrogen, Carlsbad, CA, USA). Bidirectional sequencing (using the same primers and the Sanger sequencing technique) was conducted by BaseClear (Leiden, The Netherlands). Forward and reverse reads were assembled into contigs using Geneious Prime® 2025.1.2.

### Alignment and phylogenetic analyses

A dataset of 82 ITS sequences was compiled for phylogenetic analysis. This dataset included nine newly generated sequences for this study (see Table 1) and 73 sequences publicly available in the GenBank database. The sequences were selected using a multi-step approach to ensure comprehensive coverage. To provide a robust phylogenetic context, a general set of *Lactarius* sequences representing the known genetic diversity across the three described subgenera (*L.* subg. *Lactarius*, *L.* subg. *Plinthogalus*, and *L.* subg. *Russularia*) was included. For the two newly described species, a BLAST search in GenBank was performed. The most similar and well-identified sequences from the top 100 hit results were added to the dataset. Sequences from morphologically similar and potentially closely related species from Asia were also added to the analysis to better resolve relationships. Three *Multifurca* species were chosen as an outgroup based on the results presented by Haeckel et al. (2022).

**Table 1.** Overview of specimens and sequences used, including GenBank accession numbers. Sequences in bold are newly generated for this study.

Genus species epithet	Collector/herbarium number	Country	ITS GenBank accession number
*Lactarius acris*	EU014 (UPS)	Germany	DQ421988
*Lactarius akahatsu*	JN2004-141 (GENT)	Thailand	KF133269
*Lactarius alboroseus*	SFC20150828-39	South Korea	MH985009
*Lactarius alutaceus*	SFC20120725-22	South Korea	MH984971
*Lactarius atromarginatus*	HTL314 (CMU)	Thailand	EF560674
*Lactarius atroviridis*	AV05-306 (GENT)	USA	KF133270
*Lactarius aurantiolamellatus*	Henkel 10800 (BRG, HSC, GENT) HOLOTYPE	Guyana	OM801531
*Lactarius auriolla*	RW1601 (GENT)	Sweden	KF133257
*Lactarius azonites*	DS 08-518 (GENT)	Belgium	JQ446094
*Lactarius baliophaeus*	AV05-155 (GENT)	Malawi	GU258277
*Lactarius betulinus*	SFC20150902-79 HOLOTYPE	South Korea	MH985012
*Lactarius camphoratus*	UE04.09.2004 (UPS)	Sweden	DQ422009
*Lactarius chrysorrheus*	UE04.10.2002-8 (UPS)	Italy	KF133261
*Lactarius citriolens*	UE20.09.2004-03 (UPS)	Sweden	DQ422003
*Lactarius crassiusculus*	LTH369 (GENT)	Thailand	EF560684
*Lactarius croceus*	S.D. Russell iNaturalist # 57797157	USA	OM473936
*Lactarius croceus*	S.D. Russell NAMA2018 MS iNaturalist 13904354	USA	OP541682
*Lactarius cyanotinctus*	DS06-058 (GENT)	Malaysia	GU258278
*Lactarius cyathuliformis*	UE04.09.2004-2 (UPS)	Sweden	KF133266
*Lactarius deliciosus*	JN2001-046 (GENT)	Slovakia	KF133272
*Lactarius dicymbophilus*	KM 188 (BRG, HSC, GENT)	Guyana	OM801535
*Lactarius dicymbophilus*	Henkel 10810 (BRG, HSC, GENT) HOLOTYPE	Guyana	OM801536
*Lactarius echinellus*	AV07-175 (GENT) HOLOTYPE	Sri Lanka	KF133286
*Lactarius echinus*	AV07-168 (GENT) HOLOTYPE	Sri Lanka	KF133273
*Lactarius falcatus*	KVP08-038 (GENT)	Thailand	KF133274
*Lactarius fallax*	J. Floberg-F148F (WTU)	USA	JQ446103
***Lactarius ferrugineifolius***	**DS06-261 (GENT) HOLOTYPE**	**Malaysia**	**PZ097058**
*Lactarius flexuosus*	UE06.09.2002-1 (UPS)	Sweden	DQ421992
*Lactarius fuliginosus*	MTB97-24 (GENT)	Sweden	JQ446111
***Lactarius fulvus***	**DS06-298 (GENT) HOLOTYPE**	**Malaysia**	**PZ097059**
*Lactarius gloeocarpus*	ZP-2287 (MHHNU) HOLOTYPE	China	OL770165
*Lactarius gloeocarpus*	XHW3059, KUN-HKAS 73602 (KUN)	China	OL770166
*Lactarius guyanensis*	Henkel 9630 (BRG, HSC, GENT) HOLOTYPE	Guyana	OM801539
*Lactarius guyanensis*	Henkel 9672 (BRG, HSC, GENT)	Guyana	OM801540
*Lactarius helvus*	UE08.09.2004-1 (UPS)	Sweden	KF133263
*Lactarius humiphilus*	Henkel 10784 (BRG, HSC, GENT) HOLOTYPE	Guyana	OM801544
*Lactarius humiphilus*	Henkel 10787 (BRG, HSC, GENT)	Guyana	OM801545
*Lactarius hysginus*	CNV14	USA	MT345191
*Lactarius ilicis*	E. Campo 15035 (MCVE)	Italy	JF908317
*Lactarius illyricus*	E. Campo 14644 (MCVE)	Italy	JF908315
*Lactarius incarnatus*	TPML110920-069	South Korea	MH985019
*Lactarius lignyotus*	UE06.09.2003-5 (UPS)	Sweden	DQ421993
*Lactarius lilacinus*	RW3774 (GENT)	Belgium	KF133275
*Lactarius mammosus*	UE09.09.2004-5 (UPS)	Sweden	KF133265
***Lactarius megaplinthogalus***	**AV15-111 (GENT)**	**Laos**	**PZ097060**
***Lactarius megaplinthogalus***	**AV15-108 (GENT) HOLOTYPE**	**Laos**	**PZ097061**
*Lactarius miniatescens*	AV11-177 (GENT)	Togo	KR364059
*Lactarius montoyae*	KD1065 (BSHC)	India	EF560673
*Lactarius mycenoides*	Henkel 9234 (BRG, HSC, GENT) HOLOTYPE	Guyana	MT537390
*Lactarius mycenoides*	Henkel 10798 (BRG, HSC, GENT)	Guyana	OM801550
*Lactarius novae-zelandiae*	JAC15861 (PDD)	New Zealand	MW683862
*Lactarius novae-zelandiae*	JAC13842 (PDD)	New Zealand	MW683841
*Lactarius novae-zelandiae*	JAC14702 (PDD)	New Zealand	MW683852
*Lactarius novae-zelandiae*	PDD 26527 (PDD) HOLOTYPE	New Zealand	OR348208
*Lactarius peckii*	JN2004-020 (GENT)	USA	KF133277
*Lactarius pterosporus*	KVP08-087 (GENT)	Slovenia	JQ446136
*Lactarius pubescens*	UE15.09.2002-2 (UPS)	Sweden	DQ421996
***Lactarius pudorinus***	**E4573 (GENT) ISOTYPE**	**Papua New Guinea**	**PZ097062**
*Lactarius purpureus*	FH12-008 (GENT)	Thailand	KF432966
*Lactarius quietus*	UE16.09.2004 (UPS)	Sweden	KF133264
*Lactarius romagnesii*	UE29.09.2002-6 (UPS)	France	DQ421989
***Lactarius rosascens***	**AV15-056 (GENT)**	**Laos**	**PZ097063**
***Lactarius rosascens***	**AV15-019 (GENT) HOLOTYPE**	**Laos**	**PZ097064**
*Lactarius saturnisporus*	DS07-490 (GENT)	Sri Lanka	KF133285
*Lactarius shoreae*	AV07-164 (GENT)	Sri Lanka	KF133278
*Lactarius* sp.	‘CA04’ isolate CA FUNDIS iNaturalist # 191654753	USA	PQ361817
*Lactarius* sp.	ECM root tip LH91	Malaysia	GQ268638
*Lactarius* sp.	K15052615	China	OL687393
*Lactarius* sp.	ECM root tip Se25-352	Japan	AB807952
***Lactarius* sp**.	**DS06-134 (GENT)**	**Malaysia**	**PZ097065**
*Lactarius spinosulus*	AT2003068 (UPS)	Sweden	KF133262
*Lactarius subdulcis*	JV2006-024 (GENT)	Belgium	KF133279
*Lactarius subsericatus*	UE11.10.2004-8 (UPS)	Sweden	DQ422011
*Lactarius sulphosmus*	HMAS:276808 (HMAS) HOLOTYPE	China	MG719937
*Lactarius thyinos*	A.Voitk23-08-2004 (GENT)	Canada	KF133271
*Lactarius torminosus*	RW3183 (GENT)	Czech Republic	KF133281
*Lactarius trivialis*	UE27.08.2002-17a (UPS)	Sweden	DQ421991
***Lactarius verecundus***	**DS06-032 (GENT) HOLOTYPE**	**Malaysia**	**PZ097066**
*Lactarius vietus*	UE11.19.2004-1 (UPS)	Sweden	KF133267
*Multifurca furcata*	RH7804 (NY)	Costa Rica	DQ421994
*Multifurca ochricompacta*	BB02.107 (PC)	USA	DQ421984
*Multifurca zonaria*	DED7442 (PC)	Thailand	DQ421990

This final dataset was aligned using the online version of MAFFT v7 (Katoh et al. 2018), applying the E-INS-I iterative refinement method. Phylogenetic analysis was executed with IQ-TREE2 (Trifinopoulos et al. 2016) after model selection. The selected model (using the BIC selection criterion) was TIM2e+I+G4. Credibility of the phylogenetic tree was assessed after 1,000 bootstrap replicates of the Shimodaira–Hasegawa approximate likelihood ratio test (SH-aLRT) and ultrafast bootstrap approximation (UFBoot). Values higher than 80% (SH-aLRT) and 95% (for UFBoot) were considered credible and are indicated in the phylogenetic tree. The tree was visualized in FigTree v.1.4.4 (Rambaut 2009) and further adapted for layout in Adobe Illustrator.

## Results

### Phylogeny

DNA extraction, PCR amplification, and sequencing were successful for nine collections, four of which correspond to the two new species (two per species), and the remaining five sequences correspond to additional *Lactarius* collections included to strengthen the phylogenetic framework. The ITS region was amplified and sequenced without evidence of contamination, ambiguous base calls, or intra-individual polymorphisms. Final ITS sequence lengths ranged from approximately 630 to 720 base pairs after trimming and assembly. All newly generated sequences have been deposited in GenBank (see Table 1).

BLAST searches confirmed that all sequences belong to the genus *Lactariussensu stricto*, with the highest similarity values generally below species-level thresholds for known taxa, supporting their novelty. For *Lactarius megaplinthogalus*, the closest matches corresponded to species in *Lactarius* subg. *Plinthogalus*, including *L. fulvus*, but with clear sequence divergence. Similarly, sequences of *Lactarius rosascens* showed affinity to species in *Lactarius* subg. *Lactarius* but did not match any described species with high similarity.

Sequence alignment resulted in a dataset of 82 ITS sequences with a total aligned length of approximately 750 base pairs, including gaps. No indications of sequencing artifacts, pseudogenes, or alignment ambiguities affecting phylogenetic interpretation were detected. The ITS region provided sufficient variation to distinguish both new species from all previously described taxa included in the analysis (Fig. 8).

The newly generated sequences showed high consistency within species, with minimal intraspecific variation and clear differentiation between the two taxa. These molecular results support the recognition of *Lactarius megaplinthogalus* and *Lactarius rosascens* as independent evolutionary lineages within *Lactariussensu stricto*.

### Taxonomy

#### Lactarius megaplinthogalus

Taxon classificationFungiRussulalesRussulaceae

Verbeken & Nuytinck sp. nov.

6DE1D1C4-D062-5272-AED9-92D126C4C0D6

MB862083

Figs 1, 2, 3

##### Diagnosis.

Pileus 65–110 mm diam., slightly depressed to infundibuliform, brown to blackish brown; surface soft and wrinkled. Lamellae very distant. Context first white, then turning pink, later greyish. Latex white, abundant, remarkably sticky, staining the gills pinkish, then blackish. Spores globose to subglobose, 8.7–9.7–10.7 × 8.1–8.9–9.7 µm; ornamentation composed of very irregular and interrupted ridges, locally acute and 1 to 1.8 µm high, forming an irregular and incomplete reticulum. Pleuropseudocystidia very abundant. True pleurocystidia absent. Pileipellis a trichopalisade to palisade, with terminal cells sometimes irregularly clavate, sometimes rather slender, with dark intercellular pigmentation in the upper layers.

##### Typus.

Lao PDR - Xieng Khouang Province, Pek District, Man Xom-Suea village, in mixed forest with *Fagaceae* and *Pinus kesiya*, 14 May 2015, Verbeken 15-108, HNL500819 (holotypus HNL, isotypus GENT).

##### Etymology.

referring to the stout and large fruitbodies which are outstanding in *L.* subgenus *Plinthogalus*.

##### Description.

**Figure 1.**
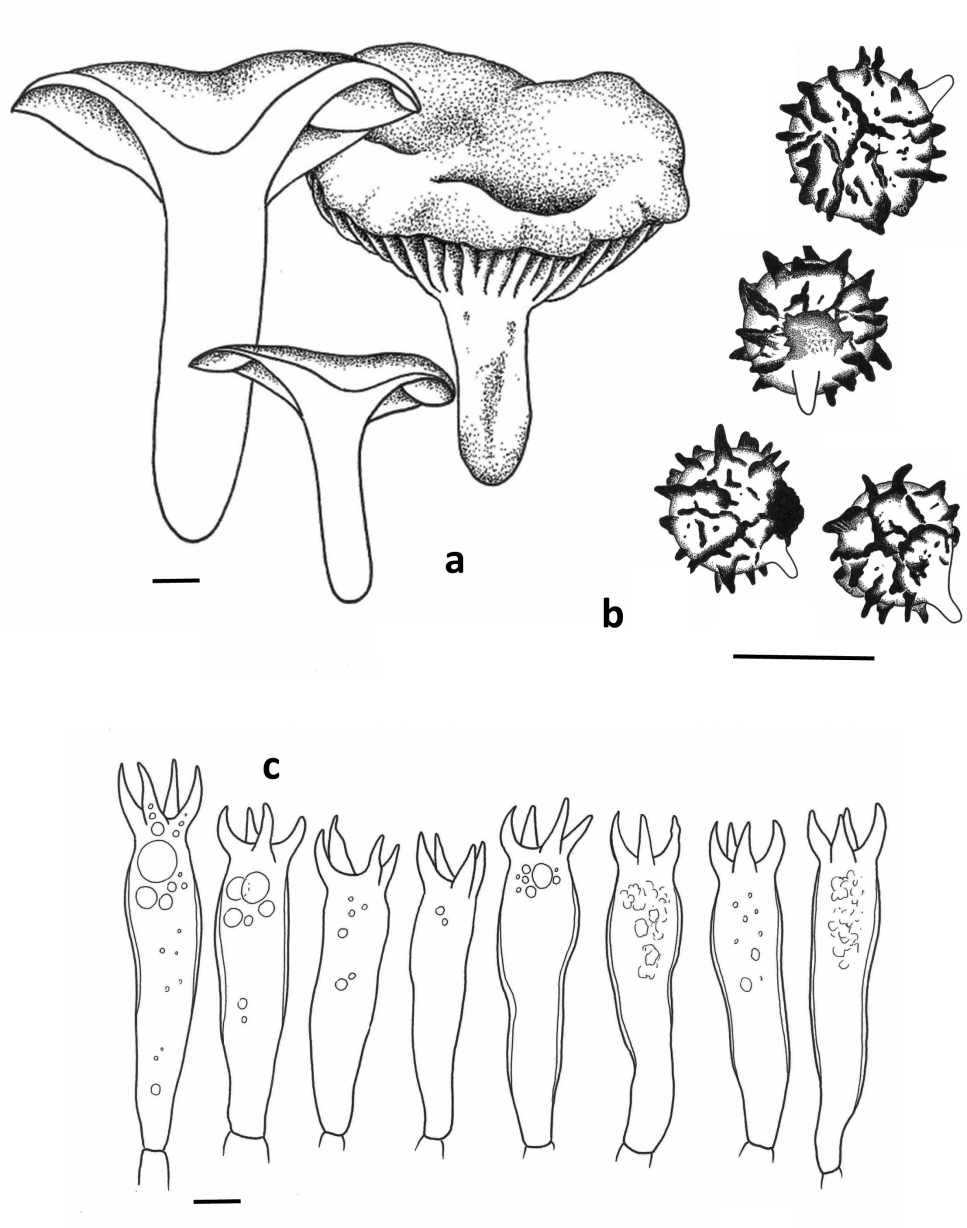
*Lactarius megaplinthogalus* (Verbeken 15-108). **a**. Basidiocarps; **b**. Basidiospores; **c**. Basidia.

**Basidiocarps** (Fig. 1a) epigeous, agaricoid, stout and firm. **Pileus** 65–110 mm diam., applanate and slightly depressed in center to broadly infundibuliform, with margin bent downwards; surface soft, dry, very wrinkled, radially wrinkled in the center, concentrically wrinkled in outer half, very dark brown, blackish brown, raw umber to coffee brown (5F7-8), in some specimens unicolorous, in other specimens locally with pale brown to whitish spots. **Stipe** 45–85 × 10–24 mm, cylindrical and regular, a bit narrower at the base; surface soft, dry, with some slight longitudinal wrinkles especially near the base, with the same greyish-brownish tinges as the pileus but much paler (5DE3-4) to almost whitish in older specimens. **Lamellae** narrowly adnexed, very distant (23 to 29 L/half a pileus, 3 L/cm) with abundant lamellulae in a regular short-long-short pattern, up to 15 mm broad, pale yellow to greyish yellow (4AB3-4), but almost whitish where they touch the stipe. **Context** thick and firm in the pileus, but thin in the outer part, solid and rather firm to compressible in the stipe, white, then changing to pink (7AB3), especially in the pileus and stipe context, later greyish, dark greyish to blackish brown, especially under the pileus and stipe surface; smell like paint; taste first agreeable and nut-like, then very acrid, and disagreeable, astringent. **Latex** white, abundant, remarkably sticky, staining the gills pinkish, then blackish. **Spore-print** cream-colored.

**Figure 2.**
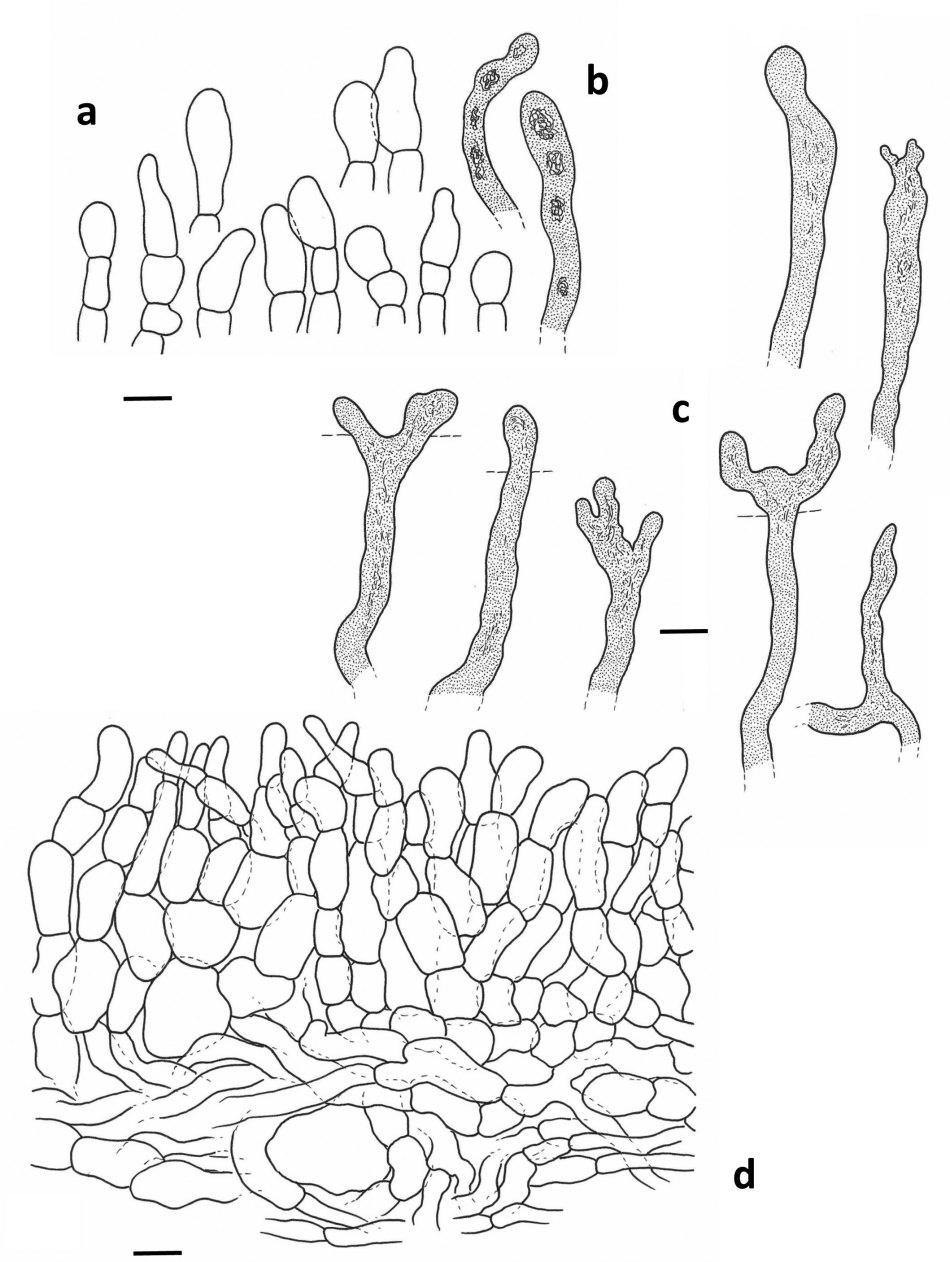
*Lactarius megaplinthogalus* (Verbeken 15-108). **a**. Marginal cells; **b**. Cheilopseudocystidia; **c**. Pleuropseudocystidia; **d**. Pileipellis halfway through the pileus – radial section.

**Spores** (Fig. 1b) globose to subglobose, 8.7–9.7–10.7 × 8.1–8.9–9.7 µm (n = 20, Q = 1.01–1.08–1.20); ornamentation amyloid, composed of very irregular and interrupted ridges, locally acute and 1 to 1.8 µm high, forming an irregular and incomplete reticulum; abundant isolated and irregular warts present: plage distally to totally amyloid. **Basidia** (Fig. 1c) subclavate, 50–75 × 10–17 µm, often remarkably thick-walled, sometimes only locally, sometimes over the whole length; sterigmata rather large and thick, up to 13 µm long; content guttulate. **Pleuropseudocystidia** (Fig. 2c) very abundant, sometimes emergent, often branching near the top, rather plump, stout and irregular, up to 10 µm diam. **True pleurocystidia** absent. **Hymenophoral trama** filled with lactifers of all diameters, very densely interwoven and branching, mixed with some normal generative hyphae; sphaerocytes absent. **Lamellar edge** completely sterile, composed of marginal cells (Fig. 2a), 10–20 × 7–12 µm, clavate to fusiform; cheilopseudocystidia (Fig. 2b) also present. **Pileipellis** (Fig. 2d) a trichopalisade to palisade, 60 to 100 µm thick; terminal cells 10–30 × 5–15 µm, sometimes irregularly clavate, sometimes rather slender, with dark intercellular pigmentation in the upper layers.

**Figure 3.**
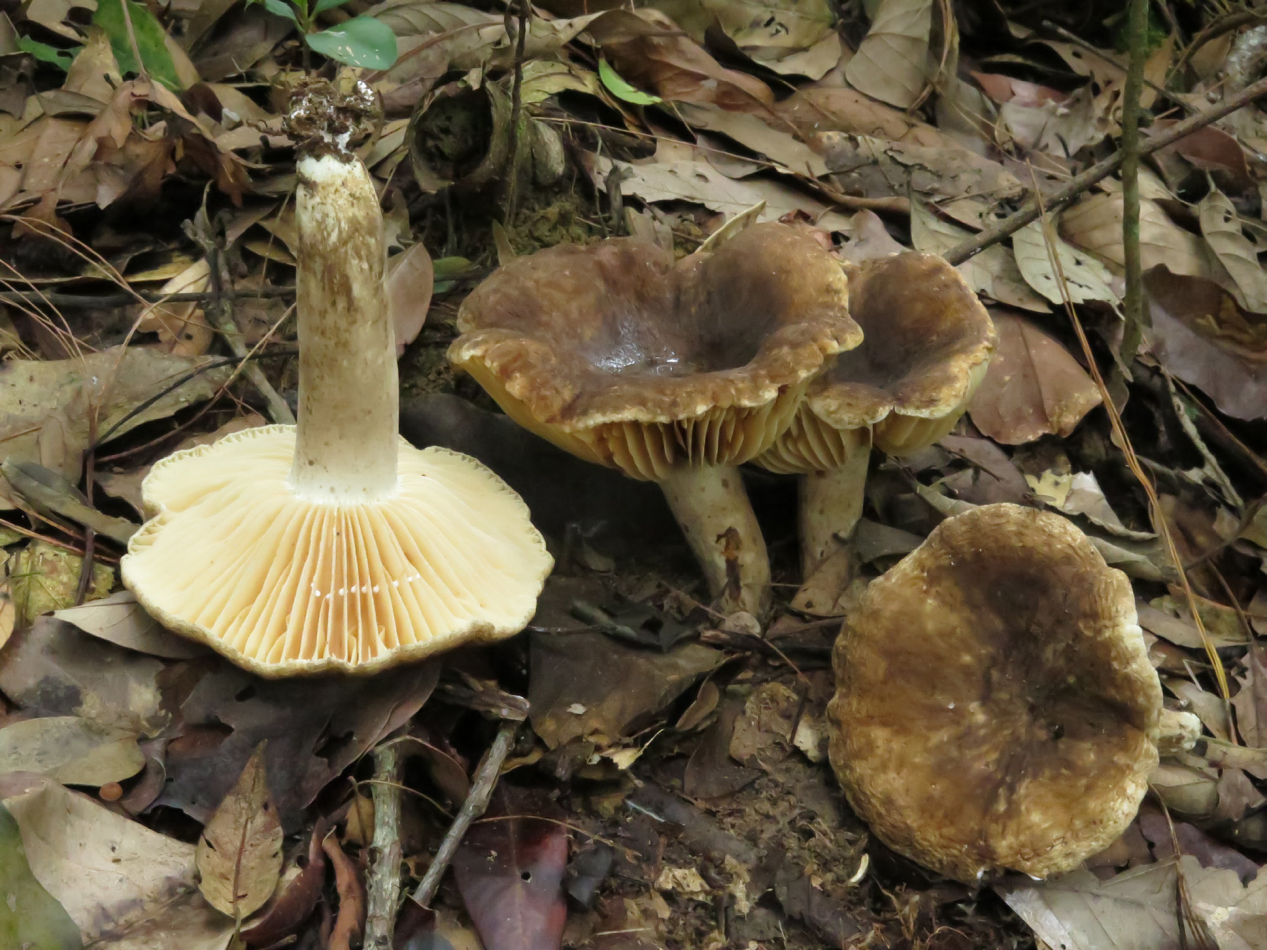
*Lactarius megaplinthogalus* (Verbeken 15-108).

##### Supplementary study material.

Lao PDR • Xieng Khouang Province, Pek District, Man Xom-Suea village; in mixed forest with *Fagaceae* and *Pinus kesiya*; 14^th^ May 2015; Verbeken 15-111 (HNL500822). • Xiang Khouang province, Phaxay district, Hai village, 19°16.00'N, 103°08.25'E (DDM), ca 1200 m.a.s.l.; in mixed forest with *Quercus* spp., *Castanea* spp., *Lithocarpus* spp., and *Pinus kesiya*; 30 May 2012; Pedersen OSP20120530–004 (HNL500350).

#### Lactarius rosascens

Taxon classificationFungiRussulalesRussulaceae

Verbeken & Nuytinck sp. nov.

737CA43A-3D90-52FE-AB8F-8E4BE909582B

MB862084

Figs 4, 5, 6, 7

##### Diagnosis.

Pileus 40–60 mm diam.; surface sticky and shiny, not slimy, yellow to honey color with darker scrobicules that form vague zones. Lamellae originally whitish, pale flesh-colored, but very soon bright pink to dark wine red by the latex. Context white, changing to pink, locally wine red to very dark; taste acrid. Latex changing to pink and wine-red. Spores subglobose to broadly ellipsoid, on average 6.5–6.8 × 5.8–6.0 µm; ornamentation with rounded ridges, up to 0.5 µm high, forming an incomplete reticulum. Pleuromacrocystidia very abundant. Pleuropseudocystidia moderately abundant. Lamellar edge fertile, with a mixture of basidia, macrocystidia, pseudocystidia and marginal cells. Pileipellis a trichoderm.

##### Typus.

Lao PDR • Xieng Khouang Province, Pek District, Dong Village, 19°29.62'N, 103°16.14'E DDM, ca 1130 m.a.s.l., in mixed forest with *Fagaceae* and *Pinus*, 10 May 2015, Verbeken 15-019, HNL500714 (holotypus HNL, isotypus GENT).

##### Etymology.

referring to turning pink.

##### Description.

**Figure 4.**
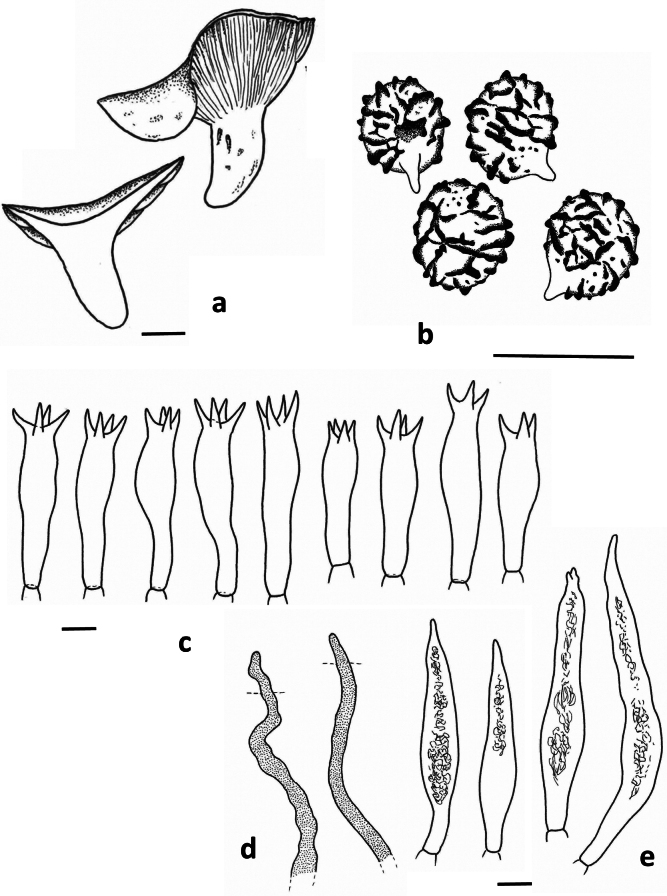
*Lactarius rosascens* (Verbeken 15-019). **a**. Basidiocarps; **b**. Basidiospores; **c**. Basidia; **d**. Pleuropseudocystidia; **e**. Pleuromacrocystidia.

**Basidiocarps** (Fig. 4a) epigeous, agaricoid, moderately large. **Pileus** 40–60 mm diam., applanate and slightly depressed in center, rather irregular, with margin a bit wavy and locally fissured; surface sticky and shiny, not slimy, yellow to honey color, butter yellow (4A5), yellowish orange (4A7), pale orange (5A3) to light orange (5A4-5, pale yellow (3A3) near margin, not really concolorous, locally more yellowish than orange with darker scrobicules that form vague zones. **Stipe** 20–30 × 8–17 mm, shortly cylindric, tapering downwards, dry, scrobiculate, off-white to yellowish (2A2, 3A2), but also stained dirty and with some pinkish shade (7A3-4). **Lamellae** adnexed to slightly decurrent and with a remarkable almost fluorescent pinkish zone where they reach the stipe, rather dense (14 L+l/cm), with abundant lamellulae of different lengths, originally whitish, pale flesh-colored, but very soon bright pink, pastel red to greyish red (7AB3-4) to dark wine red and even reddish brown (up to 8EF5) by the latex, with in this wine reddish color also a touch of darker violet, but overall strikingly pink to bordeaux. **Context** not very firm (but strongly affected by fungivorous insects in all specimens), white, changing to pink, locally wine red (7AB3-4) to very dark (8EF5) where eaten; smell not particular; taste acrid. **Latex** abundant, white, changing to pink and wine-red, staining the lamellae very dark and intense. **Spore-print** not observed.

**Figure 5.**
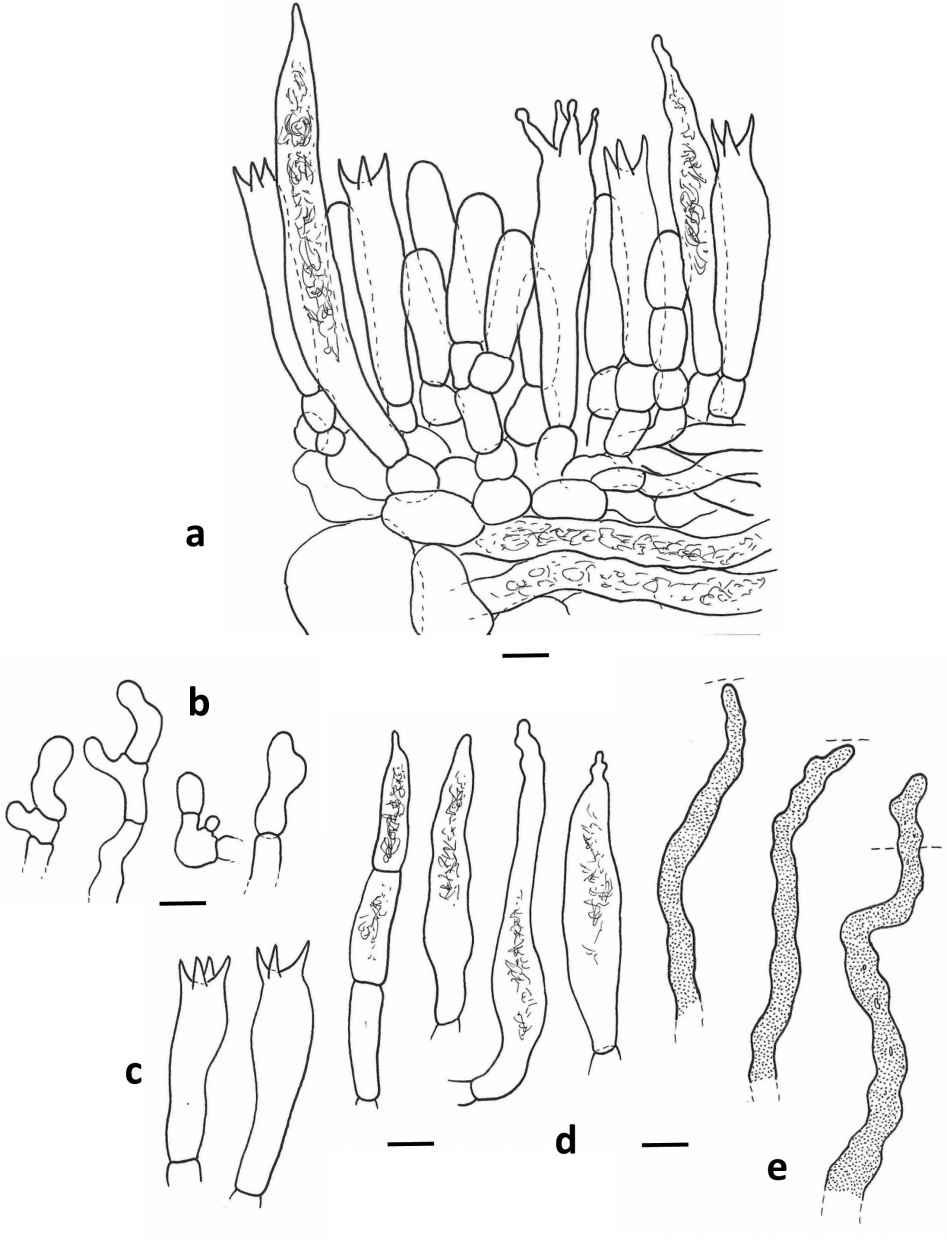
*Lactarius rosascens* (Verbeken 15-019). **a**. Hymenium; **b**. Marginal cells; **c**. Basidia at lamella edge; **d**. Cheilomacrocystidia; **e**. Cheilopseudocystidia.

**Figure 6.**
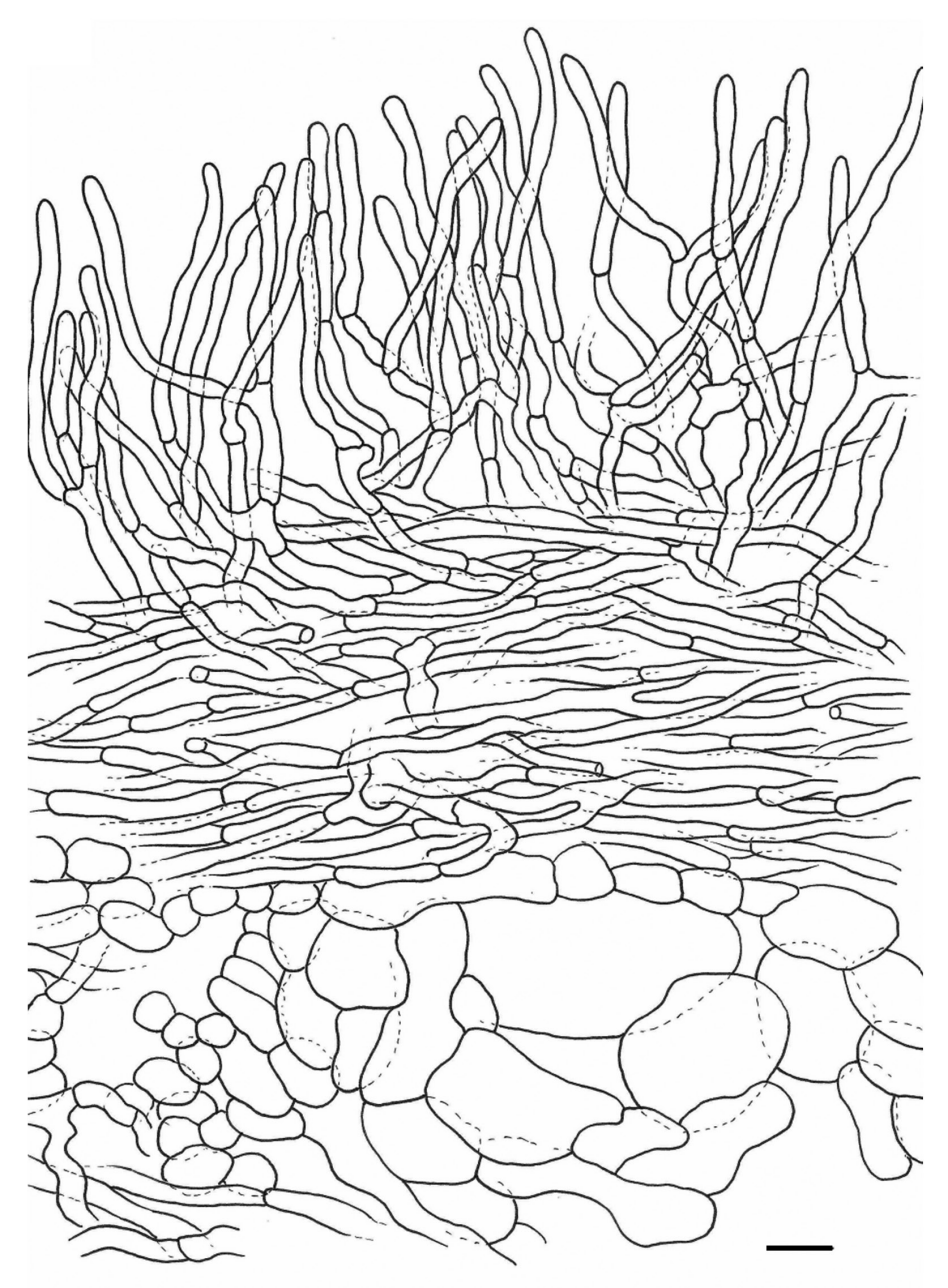
*Lactarius rosascens* (Verbeken 15-019). **a**. Pileipellis halfway through the pileus – radial section.

**Spores** (Fig. 4b) subglobose to broadly ellipsoid, 5.8–6.5–6.8–7.5 × 5.1–5.8–6.0–6.5 µm (n = 40, Q = 1.04–1.13–1.14–1.23); ornamentation amyloid, composed of rounded ridges, up to 0.5 µm high, often interrupted, forming an incomplete reticulum, some short ridges and irregular warts present; plage distally amyloid. **Basidia** (Fig. 4c, 5a) subclavate to almost cylindric, 35–45(50) × 8–10 µm, 4-spored, sometimes 3-spored; sterigmata up to 8 µm, sometimes 10 µm long. **Pleuromacrocystidia** (Fig. 4e, 5a) very abundant, emergent, 60–100 × 8–13 µm, thin-walled, fusiform, with tapering, sometimes very long, apex; apex sometimes slightly moniliform or bifurcate; content needle-like. **Pleuropseudocystidia** (Fig. 4d) moderately abundant but less common than macrocystidia, slightly emergent, narrow, 3–4 µm diam., cylindric to irregular; content opaque. **Lamellar edge** fertile, with a mixture of basidia (Fig. 5c), macrocystidia (Fig. 5d), pseudocystidia (Fig. 5e) and marginal cells (Fig. 5b); basidia present, shorter than the basidia at the sides of the lamella, up to 30–35 × 8–10 µm; cheilomacrocystidia abundant, 40–65 × 8–12 µm, fusiform with tapering and often irregular apex, sometimes remarkably septate, thin-walled, with needle-like content; cheilopseudocystidia abundant, narrow, rather irregular and twisting, with opaque content; marginal cells hyaline, thin-walled, 10–30 × 6–10 µm, shortly-septate, with terminal part subcylindric, subclavate, or very irregular, sometimes even branching. **Pileipellis** a trichoderm (Fig. 6a); subpellis a 40–60 µm thick layer of periclinally arranged hyphae; suprapellis up to 80 µm thick, composed of anticlinally arranged hyphae: terminal elements 30–40 × 2–4 µm, regularly to slightly irregularly cylindric, with rounded apex, thin-walled.

##### Supplementary study material.

Lao PDR • Xieng Khouang Province, Phoukou District, Yai Village, 19°43.90'N, 103°11.12'E DDM; in mixed forest with *Fagaceae* and *Pinus*; 11 May 2015; Verbeken 15-056, HNL500512.

**Figure 7.**
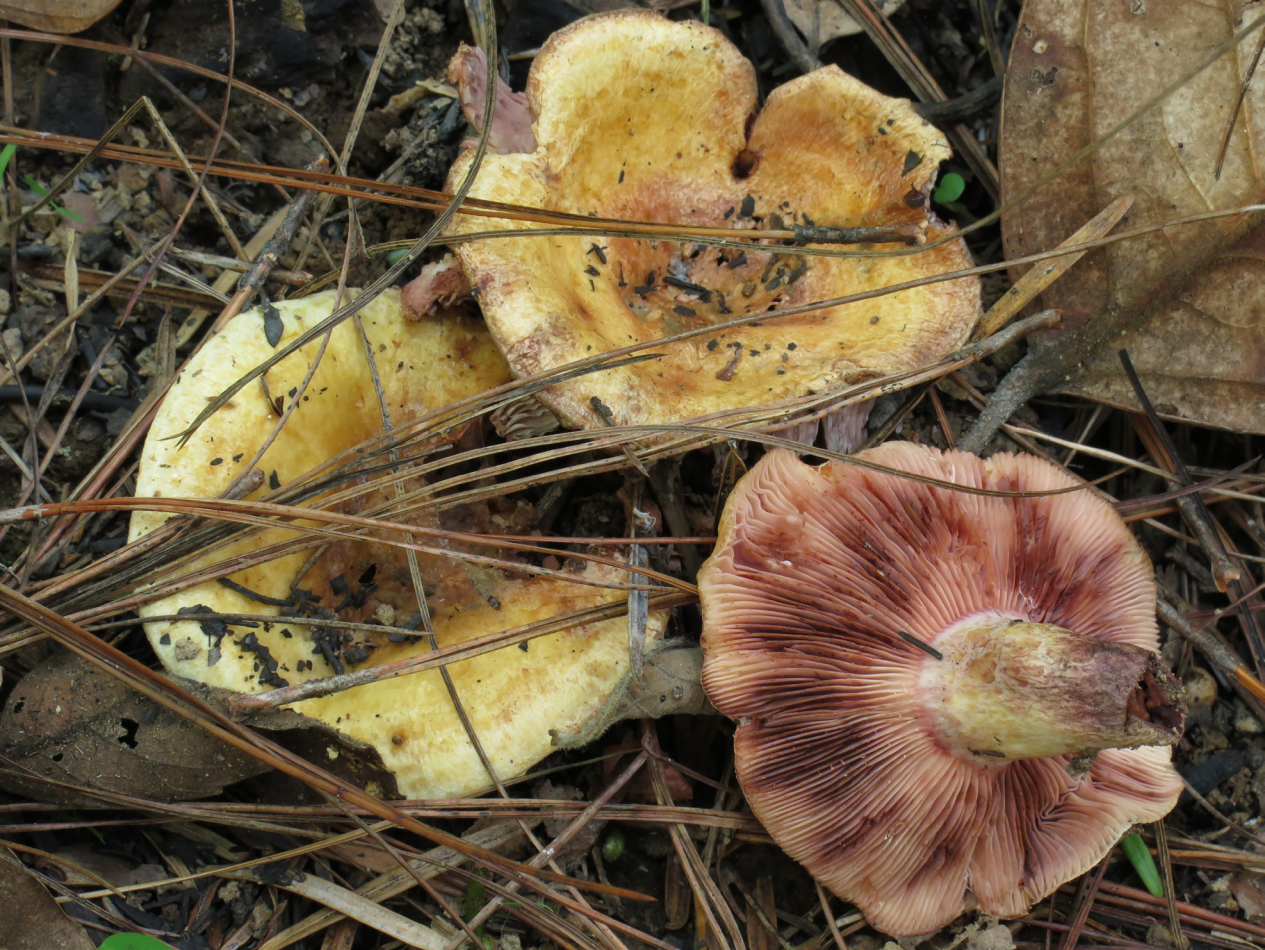
*Lactarius rosascens* (Verbeken 15-019).

## Discussion

### Lactarius megaplinthogalus

The combination of stout, dark basidiocarps with very distant, broad lamellae and a context changing to pink, later greyish, dark greyish to blackish brown, makes this species easy to recognize in the field and a very distinctive representative of *L.* subg. *Plinthogalus* sect. *Plinthogalus*. Microscopically, the distinctly thick-walled basidia are a unique feature.

The molecular analysis confirms its position in *L.* subg. *Plinthogalus*. The closest relative in the ITS tree is *Lactarius fulvus* Stubbe & Verbeken, described from lowland dipterocarp rainforest in Malaysia. It differs in the smaller, pale, dirty-ochraceous cap; latex that is turning grey with a pinkish tinge on drying; and a context that is changing to pink. The taste is only slightly acrid. The spores are comparable in size and in height of the ornamentation, but the ridges are more rounded and not as acute as in *L. megaplinthogalus* (Stubbe et al. 2008).

*Lactarius novae-zelandiae* McNabb, described from New Zealand, is a similar large and dark brown to brownish black species from this subgenus. The species differs by the lamellae that are moderately crowded and have dark brown edges, a character that was not observed in *L. megaplinthogalus*. The latex in this New Zealand species is unchanging and not staining the lamellae pinkish to blackish. The context does change to pink, salmon, and orange-red to finally vinaceous on exposure to air. The pseudocystidia are sparse and scattered. The spores are larger (9–12 × 8.2–10.5 µm) (McNabb 1971).

**Figure 8.**
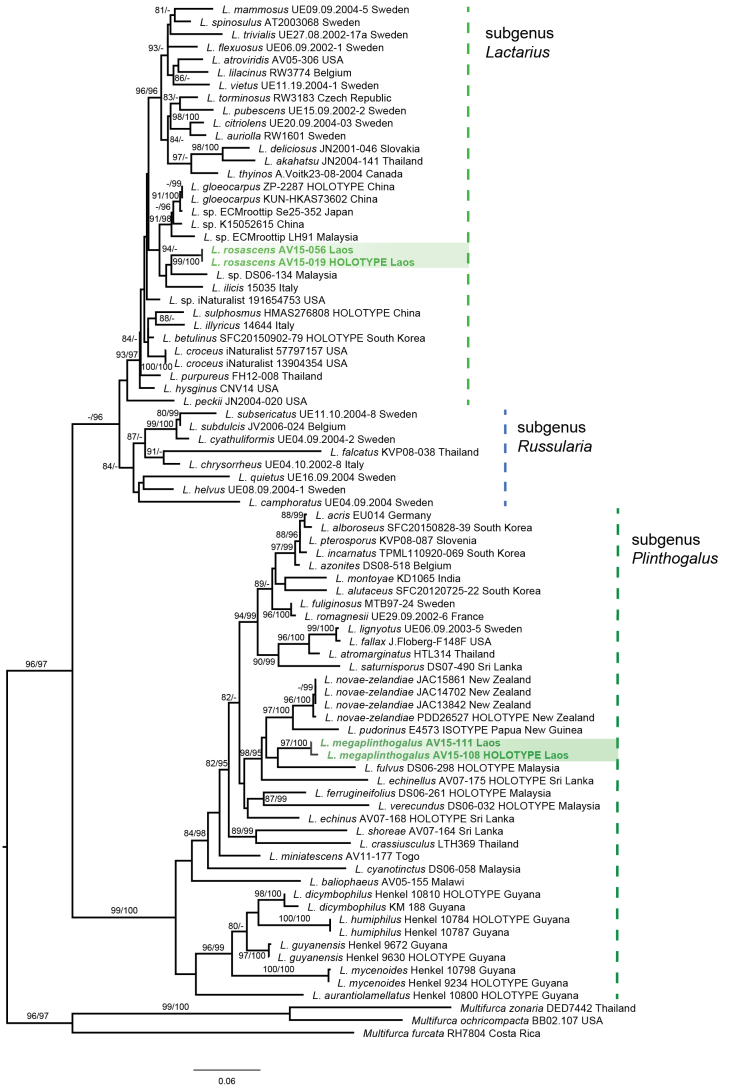
The obtained ML topology based on ITS sequences. SH-aLRT/UFBoot values are indicated if they exceed 80% and 95%, respectively. The scale bar represents the number of nucleotide changes per site.

*Lactarius pudorinus* Verbeken & Bougher was described from Papua New Guinea, growing with *Eucalyptus pellita*, *Lophostemon*, and *Melaleuca*. Like our new species proposed here, it has stout basidiocarps (with caps up to 10 cm diam.), but the cap colors are rather warm brown to pale orange. The lamellae are moderately dense, and the context changes to pink or pastel red, at most greyish red, never greyish to blackish. The basidiocarps have a distinct fishy smell when drying. Microscopically, the spores have a more distinct and regular reticulate ornamentation, and the pileipellis is a trichoderm (Verbeken et al. 2002).

*Lactarius ferrugineifolius* Stubbe & Verbeken, described from lowland dipterocarp rainforest in Malaysia, is not dark and blackish brown but has a rusty orange (5BC4–5) to orange-brown (6DEF5) cap and dull rusty orange or brownish orange lamellae that are very dark for the genus *Lactarius*. The context is unchanging when bruised or cut. The spore ornamentation is similar, but the spores are smaller (on average 7.1 × 6.4 µm) (Stubbe et al. 2008).

*Lactarius verecundus* Stubbe & Verbeken, described from the same habitat as *L. ferrugineifolius*, differs by the moderately dense and narrow lamellae and the context that is changing salmon pink, never greyish to blackish. The spores (on average 6.6 × 6.1–6.2 µm) are strikingly winged and zebroid, and the ornamentation is up to 2 µm high. The pileipellis is a trichoderm (Stubbe et al. 2008).

*Lactarius echinus* Stubbe & Verbeken and *L. echinellus* Verbeken & Stubbe are also quite closely related, but they are gasteroid, so morphologically very different (Verbeken et al. 2014b).

Summarized, *Lactarius megaplinthogalus* represents a remarkable addition to *L.* subg. *Plinthogalus*. Its large, stout, dark-colored basidiocarps, extremely distant lamellae, and strongly discoloring, sticky latex give it a very characteristic field appearance. The combination of these macromorphological traits with the presence of distinctly thick-walled basidia is unique within the subgenus.

### Lactarius rosascens

At first sight this species reminds a representative of *L.* sect. *Deliciosi* because of the scrobicules and all these bright colors and color changes, but the milk is completely white when exuded. There are some species in the section with white milk (*L. porninsis* Rolland, *L. splendens* Hesler & A.H. Sm., *L. aurantiozonatus* H. Lee, Wisitr. & Y.W. Lim, and *L. mundus* X.H. Wang & S.Q. Cao), but there the milk is unchanging and not at all followed by spectacular color changes that are so typical for the *Deliciosi* representatives. Microscopically, the pileipellis structure is very striking: a very well-developed trichoderm is rare in *L.* subg. *Lactarius*.

The closest identified BLAST hit in the same geographical area leads us to *Lactarius gloeocarpus* Fang Wu, X.H. Wang & Z.H. Chen, known from forests dominated by *Pinus taiwanensis* and fagaceous trees in central-southern China (Hunan and Jiangxi) and Japan (Wu et al. 2022). This species shares the remarkable trichoderm structure in the pileipellis, although here it is an ixotrichoderm with more shrivelled hyphae embedded in a thick slime layer. The pileus is pale yellow to light yellow, sometimes with a greenish tinge, but spectacular discolorations, as in our new species from Laos, are lacking, though the lamellae are turning brownish when bruised. However, context and latex are unchanging.

Most potentially related species, suggested by our ITS phylogeny, can be readily excluded based on differences in pileus and latex color, as well as the absence of comparable color changes. The color combination observed in *Lactarius rosascens*—a yellow to honey-colored, scrobiculate pileus combined with an abundant white latex that rapidly turns bright pink to deep wine red and intensely stains the lamellae—is exceptional within *Lactarius*.

Species such as *L. purpureus* R. Heim (from Asia) and *L. betulinus* H. Lee, Wisitr. & Y.W. Lim (from South Korea) differ markedly in having darker, more uniformly brown to purplish pilei and latex that is either unchanging or only weakly discoloring, never producing the vivid pink to wine-red reactions seen in *L. rosascens*. The hypogeous, gasteroid *L. sulphosmus* G.J. Li & R.L. Zhao is morphologically and ecologically distinct and lacks conspicuous latex color changes altogether (Li et al. 2018).

European species, including *L. illyricus* Piltaver, *L. hysginus* (Fr.) Fr., and *L. ilicis* Sarnari, also differ by their generally darker, reddish-brown to vinaceous pilei and latex that remains white or only slowly browning, without the rapid and intense pink to wine-red discoloration characteristic of *L. rosascens*. None of these species show the striking staining of the lamellae observed in the new taxon.

The North American *L. croceus* Burl. can likewise be excluded by its orange- to saffron-colored pileus and latex that does not undergo pronounced pink or wine-red color changes.

Taken together, *Lactarius rosascens* is equally distinctive and easily recognizable by its vivid color reactions. The combination of a yellow to honey-colored, scrobiculate pileus with an abundant white latex that rapidly turns bright pink to deep wine red and intensely stains the lamellae appears to be unparalleled within *Lactarius*. Although its bright colors and scrobiculate pileus may recall species of *L.* sect. *Deliciosi* at first glance, the trichodermal pileipellis structure and phylogenetic placement tell a different story. For the moment it is not possible to classify the species at a sectional level.

### General

Southeast Asia is widely recognized as a major center of fungal diversity, yet large parts of the region remain insufficiently explored from a mycological perspective (Mueller et al. 2007; Stallman et al. 2024; Hyde et al. 2024; Hongsanan et al. 2025; Nie et al. 2025; Zhao et al. 2026). Laos, in particular, represents a largely untapped reservoir of fungal diversity due to its high forest cover, complex topography, and wide range of forest types supporting diverse ectomycorrhizal host trees (PBSAP 2013; MoNRE 2016; MAF 2021).

The discovery of *Lactarius megaplinthogalus* and *L. rosascens* highlights both the high diversity and the distinctiveness of *Lactarius* lineages occurring in montane forests of northern Laos. Both species were collected in mixed forests dominated by *Pinaceae* and *Fagaceae*, host families known to support rich ectomycorrhizal fungal communities throughout Southeast Asia (Wang et al. 2012, 2025; Verbeken et al. 2014a, 2014b). These findings are consistent with broader regional studies showing that tropical and subtropical Asian forests harbor highly diverse and often endemic milkcap assemblages, many of which belong to lineages distinct from their temperate counterparts (Wisitrassameewong et al. 2014a, 2014b, 2015; De Crop et al. 2018a, 2018b; Huang et al. 2022; Tang et al. 2022).

The recognition of the two new species is supported by a combination of highly distinctive morphological characters and molecular phylogenetic evidence based on ITS sequences. The ITS region is widely accepted as the primary DNA barcode for fungi due to its high species-level resolution and extensive representation in reference databases (White et al. 1990; Gardes and Bruns 1993). In *Lactarius* and related genera, ITS has proven highly effective for species delimitation and phylogenetic placement, particularly when combined with detailed morphological observations (Verbeken et al. 2014a, 2014b; De Crop et al. 2018a, 2018b; Wang et al. 2025). In the present study, ITS sequences clearly resolve both taxa as well-supported and independent evolutionary lineages within *Lactariussensu stricto*.

At the same time, the precise infrageneric placement of the new taxa, particularly *Lactarius rosascens*, remains uncertain. While *L. megaplinthogalus* can be confidently assigned to *Lactarius* subg. *Plinthogalus*, sect. *Plinthogalus*, the sectional placement of *L. rosascens*, cannot yet be resolved. This reflects broader challenges in the infrageneric classification of *Lactarius*, especially in tropical regions where numerous lineages remain undescribed and phylogenetic frameworks are still incomplete (Verbeken and Nuytinck 2013; De Crop et al. 2018a, 2018b; Wang et al. 2025). Resolving these relationships will require comprehensive multilocus phylogenetic analyses combined with expanded taxon sampling across tropical Asia, an effort that is currently underway but beyond the scope of the present study.

Beyond their taxonomic significance, the discovery of these two species underscores the importance of continued fungal exploration in under-sampled tropical regions. Even highly conspicuous macrofungi with large and distinctive fruitbodies, such as the species described here, can remain undocumented for long periods, illustrating the magnitude of undescribed fungal diversity (Hyde et al. 2024; Hongsanan et al. 2025; Nie et al. 2025).

## Supplementary Material

XML Treatment for Lactarius megaplinthogalus

XML Treatment for Lactarius rosascens

## Additional information

### Use of AI

No use of AI was reported.

### Author ORCIDs

Annemieke Verbeken https://orcid.org/0000-0002-6266-3091

Ole S. Pedersen https://orcid.org/0009-0000-1271-4607

Andy F. S. Taylor https://orcid.org/0000-0003-0727-8171

Jorinde Nuytinck https://orcid.org/0000-0001-9250-377X

### Data availability

All of the data that support the findings of this study are available in the main text.
